# Supplementary material for: Electroactive materials with tunable response based on block copolymer self-assembly
Source: Nat Commun. 2019 Feb 5;10:601. doi: 10.1038/s41467-019-08436-2 (PMC6363725; doi:10.1038/s41467-019-08436-2)
Supplement: Supplementary file 1 — Supplementary Information [file 41467_2019_8436_MOESM1_ESM.pdf]

Supplementary Information File

Electroactive materials with tunable response based on block copolymer self-assembly

Terzic et al.

Supplementary Note 1. Calculation of the TrFE content inside P(VDF-TrFE)

Supplementary Note 2. ^{19}F NMR

Supplementary Note 3. Calculation of P2VP and PS weight fraction inside block copolymers

Supplementary Note 4. End group exchange

Supplementary Note 5. Thermal annealing at 120 ºC in paraelectric phase

Supplementary Note 6. WAXS of P(VDF$_{50}$-TrFE$_{50}$)

Supplementary Note 7. *D-E* loops of P2VP-*b*-P(VDF$_{70}$-TrFE$_{30}$)-*b*-P2VP crystallized from melt

Supplementary Note 1. Calculation of the TrFE content inside P(VDF-TrFE)

The molar ratio between VDF and TrFE was determined using ^{1}H NMR spectra from following equations:

$$\%VDF = \frac{n_{VDF}}{n_{VDF}+n_{TrFE}} \tag{1}$$

$$\%TrFE = \frac{n_{TrFE}}{n_{VDF}+n_{TrFE}} \tag{2}$$

Where :

$$n_{TrFE} = \int_{5}^{6} CF\boldsymbol{H}$$

$$n_{VDF} = \frac{1}{2} \times \left(\int_{2.9}^{3.4} VDF_{head-tail} + \int_{2.3}^{2.6} VDF_{head-head} \right)$$

Supplementary Note 2. ^{19}F NMR

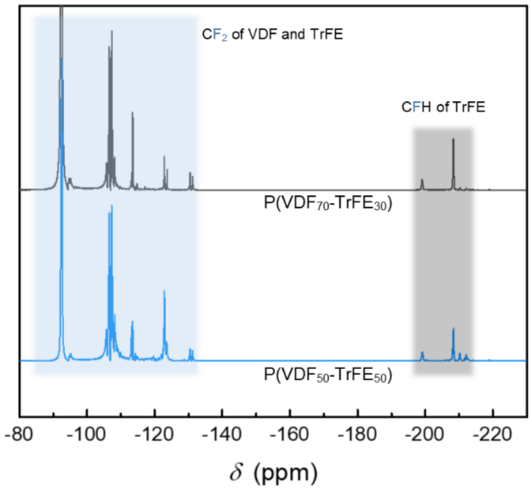

Supplementary Figure 1. ^{19}F NMR spectra. ^{19}F NMR spectra in acetone-*d6* of chlorine terminated P(VDF_{70}-$TrFE_{30}$) and P(VDF_{50}-$TrFE_{50}$).

Supplementary Note 3. Calculation of P2VP and PS weight fraction inside block copolymers

The weight fraction *f* of P2VP and PS inside block copolymers is calculated using ^{1}H-NMR of block copolymers comparing the ratio between the aromatic peak(s) of P2VP and PS and peak that corresponds to TrFE units in copolymer (molar content of TrFE units inside copolymer is previously determined as described in Supplementary Note 1).

$$f_{PS} = \frac{\frac{1}{5} I_{PS}{}_{6}^{8} \times M_{Styrene}}{\frac{1}{5} I_{PS}{}_{6}^{8} \times M_{Styrene} + I_{TrFE}{}_{5}^{6} \times M_{TrFE} + \frac{n_{VDF}}{n_{TrFE}} \times I_{TrFE}{}_{5}^{6} \times M_{VDF}} \tag{3}$$

$$f_{P2VP} = \frac{\frac{1}{4} I_{P2VP}{}_{6}^{9} \times M_{2VP}}{\frac{1}{4} I_{P2VP}{}_{6}^{9} \times M_{2VP} + I_{TrFE}{}_{5}^{6} \times M_{TrFE} + \frac{n_{VDF}}{n_{TrFE}} \times I_{TrFE}{}_{5}^{6} \times M_{VDF}} \tag{4}$$

Supplementary Note 4. End group exchange

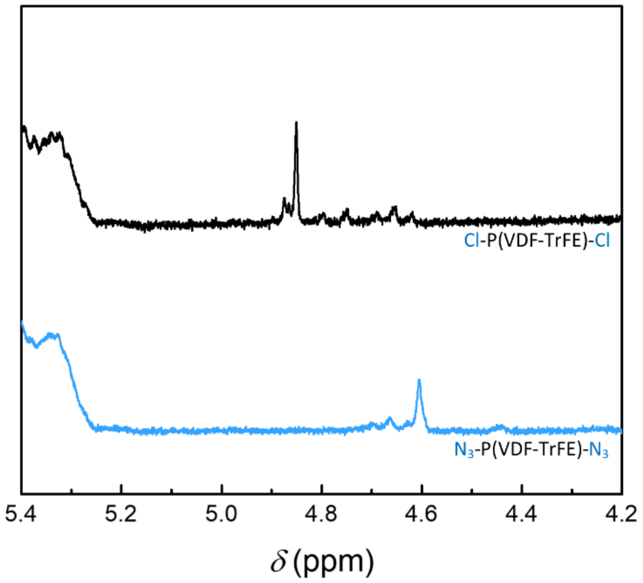

Supplementary Figure 2. End group exchange. ^{1}H NMR spectra in DMSO-d_6 of chlorine and azide terminated P(VDF-TrFE). The shift of the methylene proton from 4.85 to 4.60 ppm is a consequence of the full substitution of end groups from chlorine to azide, after stirring chlorine terminated polymer with NaN_3 in dimethylformamide overnight.

Supplementary Note 5. Thermal annealing at 120 ºC in paraelectric phase

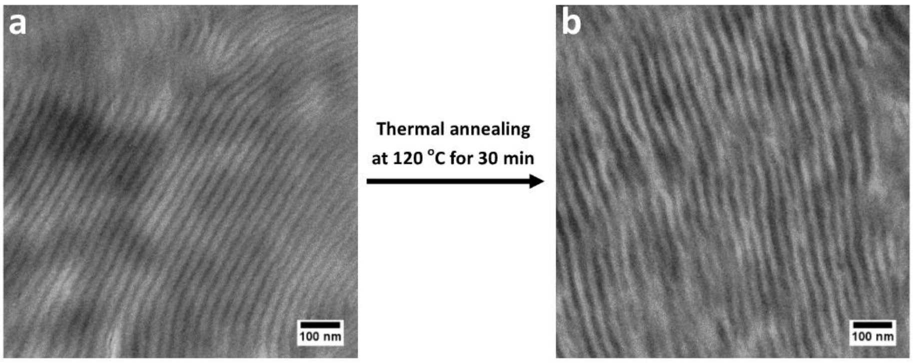

Supplementary Figure 3. Thermal annealing of block copolymer in paraelectric phase. TEM images of P2VP-*b*-P(VDF$_{70}$-TrFE$_{30}$)-*b*-P2VP a) after solvent casting and b) after thermal annealing in paraelectric phase at 120 ºC.

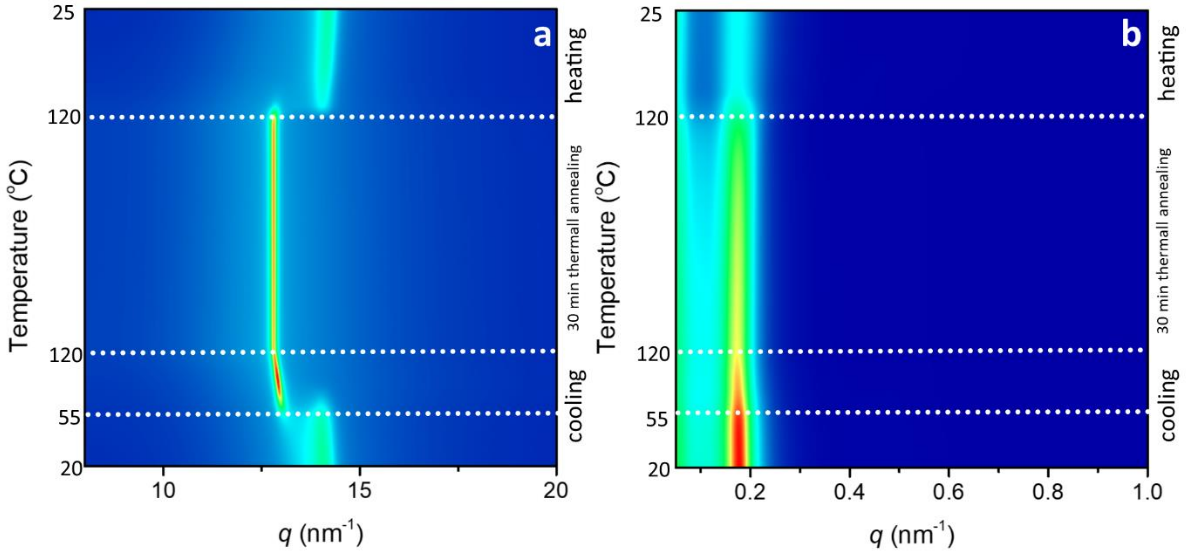

Supplementary Figure 4. Structural changes of block copolymer during thermal annealing in paraelectric phase. Temperature resolved a) WAXS and b) SAXS profiles of PS-*b*-P(VDF$_{70}$-TrFE$_{30}$)-*b*-PS during annealing in the paraelectric phase at 120 ºC for 30 min. The sample is heated/cooled at a heating/cooling rate 10 ºC min^{-1}. The same findings are obtained for P2VP-*b*-P(VDF$_{70}$-TrFE$_{30}$)-*b*-P2VP block copolymers.

Supplementary Note 6. WAXS of P(VDF_{50}-$TrFE_{50}$)

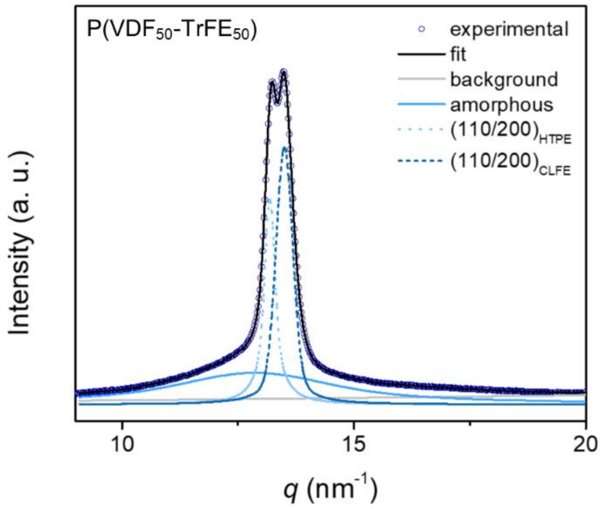

Supplementary Figure 5. Crystalline phase of P(VDF_{50}-$TrFE_{50}$). WAXS profiles of P(VDF_{50}-$TrFE_{50}$). Peak fitting is performed to determine the crystalline phases and overall crystallinity of the polymer samples. The experimental profiles were deconvoluted by using the sum of a linear background, and few pseudo-Voigt peaks describing the scattering from the amorphous and the different crystalline phases

Supplementary Note 7. *D-E* loops of P2VP-*b*-P(VDF_{70}-$TrFE_{30}$)-*b*-P2VP crystallized from melt

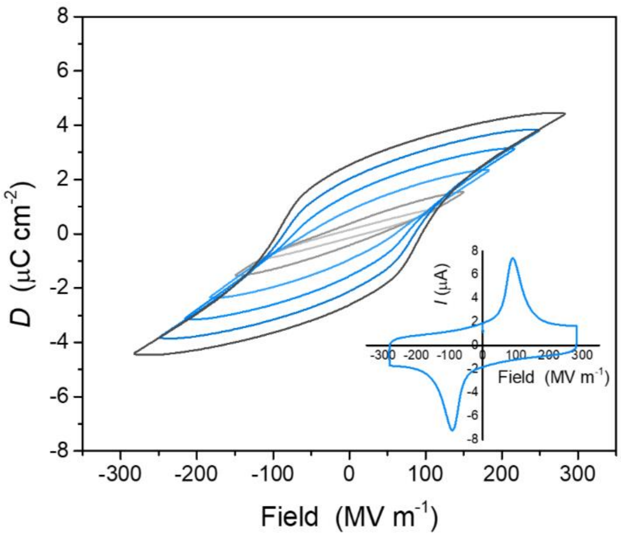

Supplementary Figure 6. Ferroelectric hysteresis loops. Bipolar *D*-*E* loops for P2VP-*b*-P(VDF_{70}-$TrFE_{30}$)-*b*-P2VPcrystallized from the melt. The data is obtained using triangular waveform at frequency of 10 Hz.
